# *Streptococcus suis* outbreak caused by an emerging zoonotic strain with acquired multi-drug resistance in Thailand

**DOI:** 10.1099/mgen.0.000952

**Published:** 2023-02-15

**Authors:** Jaime Brizuela, Rattagan Kajeekul, Thomas J. Roodsant, Athita Riwload, Parichart Boueroy, Auttapong Pattanapongpaibool, Janjira Thaipadungpanit, Piroon Jenjaroenpun, Thidathip Wongsurawat, Elizabeth M. Batty, Boas C. L. van der Putten, Constance Schultsz, Anusak Kerdsin

**Affiliations:** ^1^​ Amsterdam UMC location University of Amsterdam, Department of Global Health, Amsterdam Institute for Global Health and Development, Paasheuvelweg 25, Amsterdam, The Netherlands; ^2^​ Amsterdam UMC location University of Amsterdam, Department of Medical Microbiology and Infection Prevention, Meibergdreef 9, Amsterdam, The Netherlands; ^3^​ Department of Medicine, Maharat Nakhon Ratchasima Hospital, Nakhon Ratchasima, Thailand; ^4^​ Clinical Microbiology Laboratory, Department of Medical Technology, Maharat Nakhon Ratchasima Hospital, Nakhon Ratchasima, Thailand; ^5^​ Faculty of Public Health, Kasetsart University Chalermphrakiat Sakon Nakhon Province Campus, Sakon Nakhon, Thailand; ^6^​ Department of Clinical Tropical Medicine, Faculty of Tropical Medicine, Mahidol University, Bangkok, Thailand; ^7^​ Division of Bioinformatics and Data Management for Research, Department of Research and Development, Faculty of Medicine Siriraj Hospital, Mahidol University, Bangkok, Thailand; ^8^​ Mahidol-Oxford Tropical Medicine Research Unit, Faculty of Tropical Medicine, Mahidol University, Bangkok, Thailand; ^9^​ Centre for Tropical Medicine and Global Health, Nuffield Department of Medicine, University of Oxford, Oxford, UK

**Keywords:** antimicrobial resistance, outbreak, *Streptococcus suis*, whole genome sequencing, zoonosis

## Abstract

*
Streptococcus suis* is an emerging zoonotic swine pathogen which can cause severe infections in humans. In March 2021, an outbreak of *S. suis* infections with 19 confirmed cases of septicemia and meningitis leading to two deaths, occurred in Nakhon Ratchasima province, Thailand. We characterized the outbreak through an epidemiological investigation combined with Illumina and Nanopore whole genome sequencing (WGS). The source of the outbreak was traced back to a raw pork dish prepared from a single pig during a Buddhist ceremony attended by 241 people. WGS analysis revealed that a single *S. suis* serotype 2 strain belonging to a novel sequence type (ST) of the emergent Thai zoonotic clade CC233/379, was responsible for the infections. The outbreak clone grouped together with other Thai zoonotic strains from CC233/379 and CC104 in a global *S. suis* phylogeny and capsule switching events between serotype 2 zoonotic strains and serotype 7 porcine strains were identified. The outbreak strain showed reduced susceptibility to penicillin corresponding with mutations in key residues in the penicillin binding proteins (PBPs). Furthermore, the outbreak strain was resistant to tetracycline, erythromycin, clindamycin, linezolid and chloramphenicol, having acquired an integrative and conjugative element (ICE) carrying resistance genes *tetO* and *ermB,* as well as a transposon from the IS1216 family carrying *optrA* and *ermA*. This investigation demonstrates that multi-drug resistant zoonotic lineages of *S. suis* which pose a threat to human health continue to emerge.

## Data Summary

The supplementary figures and tables can be found in the online version of this article. Raw Illumina and Nanopore FastQ sequences can be found in the NCBI Short Read Archive under the BioProject PRJNA853715. Genome assemblies have been deposited in Genbank and are available under the same BioProject. A list containing the SRA, Genbank and Biosample accession numbers of genomes generated in this study and public genomes used in this study can be found in the supplementary material (Table S1, available in the online version of this article).

### Outcome

In this study, we characterized the outbreak of *S. suis* infections that occurred in Nakhon Ratchasima province during March–April 2021. This outbreak demonstrates the public health threat that *S. suis* poses in Thailand. In an epidemiological investigation, we determined that the outbreak originated from a single source, which was a multi-drug resistant *S. suis* strain, associated with consumption of a raw pork dish prepared from a single pig. In a phylogenetic analysis of whole genome sequences of outbreak and unrelated strains, we show that the outbreak *S. suis* strain has a novel sequence type (ST) that belongs to the zoonotic lineage CC233/379. The zoonotic CC233/379 clade emerged in Thailand in the last two decades and this is the first report of an outbreak of human infections caused by *S. suis* belonging to this clade. We linked the antimicrobial resistance profile to key mutations and the acquisition of multiple mobile genetic elements. In conclusion, a multi-drug resistant *S. suis* strain which belongs to an emerging zoonotic clade caused an outbreak of human infections with high morbidity and mortality.

## Introduction

*Streptococcus suis* is an important zoonotic pathogen which causes invasive infections in both humans and pigs resulting in severe cases of meningitis, arthritis, and septicemia. Human infections occur primarily through close contact with infected pigs or through consumption of raw or undercooked contaminated pork products [1, 2]. The emergence of zoonotic *S. suis* appears to date back to the early twentieth century [3]. Since then, *S. suis* has caused two large zoonotic outbreaks in China during 1998 and 2005 with high mortality rates (56 and 18% respectively) and is the leading cause of bacterial meningitis in adult patients in Thailand, Vietnam, and Hong Kong [4–6]. Infections in Europe and North America are mostly an occupational hazard [7]. The increased reporting of human cases paired with a high morbidity have marked *S. suis* as a major public health threat, especially in China and South-East Asia [5].

In *S. suis *, there are 29 recognized serotypes and high levels of capsular recombination have been observed [3]. While some serotypes are more predominant in certain lineages, most lineages display several serotypes, and each serotype can often be found across multiple lineages [3]. Human infections are mainly restricted to serotype 2 and to a lesser extent serotype 14, and both capsular polysaccharides are structurally similar, sharing an identical side chain terminated with the sialic acid Neu5Ac [8, 9]. Isolated cases of human infections caused by other serotypes including 4, 5, 9, 14, 16, 21, 24, and 31 have also been reported [10–17]. In contrast, the *S. suis* population that is associated with invasive disease in pigs is more diverse, displaying a wider range of serotypes of which 1, 1/2, 2, 3, 5, 7, 9 and 14 are predominant, indicating that a certain degree of host-pathogen adaptation is required for virulence [18, 19]. Serotype 2 strains of sequence types (ST) that belong to Clonal Complex 1 (CC1) have spread worldwide and are the leading cause of human invasive infections [20]. Human infections caused by *S. suis* strains belonging to other clonal complexes such as CC20, CC25, CC28, and CC104 have also been reported [20]. Some of these clonal complexes appear to be limited to certain geographic areas, for instance, human infections caused by serotype 2 CC20 *S *. *suis* strains have only been reported in the Netherlands [21].

The *S. suis* population in Thailand is particularly interesting due to the occurrence of highly diverse zoonotic isolates in terms of both serotypes and STs. While most human infections are still caused by serotype 2 strains, a small subset of reported cases are caused by serotype 14 strains (4.5–14.1 % varying between studies) and sporadic infections by isolates of serotypes 4, 5, 9, 24 and 31 have also been reported [2, 13, 22]. In terms of ST profiles, more than 50% of the reported zoonotic cases were caused by isolates belonging to CC1. However, CC104 and CC233/379, two emergent zoonotic lineages which have only been reported from Thailand, are responsible for ~37 % of Thai human infections [22]. These two clades have remained largely uncharacterized until this date. Additionally, sporadic zoonotic infections caused by strains belonging to CC25, CC28, CC221/234 and CC94 have also been reported in Thailand [23]. Moreover, Thai *S. suis* strains display a broad range of antimicrobial resistance (AMR) profiles against multiple antibiotic classes, with tetracycline and macrolide–lincosamide–streptogramin B (MLS_B_) resistance being widespread across Thai *S. suis* isolates [24, 25].

An outbreak of human infections with *S. suis* occurred in Northeast Thailand in March-April, 2021. A total of 88 suspected cases were involved, of which, 19 cases were confirmed and two patients died. In the present study, we combined clinical and molecular epidemiology to characterize this *S. suis* outbreak.

## Methods

### Outbreak investigation

Epidemiologists from the Nakhon Ratchasima Public Health Office investigated the outbreak following a report from the Maharaj Nakhon Ratchasima hospital on April 1^st^, concerning the death of the first of two patients with a history of raw pork consumption during an ordination ceremony, who died from a *S. suis* infection. In parallel, the death of the two patients was reported in the local news, which prompted 199 participants of the ordination ceremony to report at the community hospital between April 3^rd^ and April 8^th^. The epidemiologists reviewed the patients’ information, interviewed the patients, and conducted a survey amongst ceremony participants using a questionnaire. Finally, an attending veterinarian investigated the farm from which the contaminated pork originated and interviewed the farmer [26].

Suspected cases of *S. suis* infection were defined as: any patient who attended the ordination ceremony on March 28^th^, had a history of contact with food items served during the ceremony, and who displayed any of the following clinical symptoms: headache, muscle pain, chills, joint pain, stomach ache, diarrhoea, vomiting or nausea.

Blood cultures were taken from all suspected cases (*n*=88). Cerebrospinal fluid (CSF) was extracted by lumbar puncture for patients showing symptoms of meningitis (*n*=3). Cases were confirmed following positive blood and/or CSF cultures. Presumptive identification of *S. suis* was carried out using ViTek-2 in the Maharaj Nakhon Ratchasima hospital as well as by a previously described *S. suis* typing PCR [26–28]. All isolates were stored at −80^ ^°C for further analysis.

### Clinical investigation

Medical records of 19 confirmed outbreak cases and seven epidemiologically-unrelated post-outbreak cases which occurred in May 2021 were reviewed retrospectively by attending physicians at the Maharaj Nakhon Ratchasima hospital in Thailand using the clinical case record form, which was approved by the Ethics Committee of the hospital. The clinical manifestations of *S. suis* infections were classified according to criteria described elsewhere [22]. *S. suis *-associated sepsis was defined as any confirmed case displaying Systemic Inflammatory Response Syndrome (SIRS) [29].

### Bacterial strains

Out of 19 confirmed outbreak cases, 15 isolates could be retrieved for multi-locus sequence typing (MLST) and whole genome sequencing (WGS) and were included in the analysis. These isolates were supplemented with seven isolates obtained from seven epidemiologically-unrelated post-outbreak cases of human *S. suis* infection, as described above, for comparison.

### Multi-locus sequence typing (MLST)

MLST was performed as described previously [30]. In brief, the seven housekeeping genes *aroA, cpn60, dpr, gki, mutS, recA* and *thrA* were amplified and Sanger sequenced at Apical Scientific (Seri Kembangan, SGR, Malaysia) on a 96-capillary 3730xl DNA Analyser (Applied Biosystems, Waltham, MA, USA). MLST alleles, resulting sequence type (ST), and clonal complex (CC) were assigned using the *S. suis* MLST database, which can be accessed at https://pubmlst.org/ssuis/.

### Whole-genome sequencing and assembly

*S. suis* isolates were grown on sheep blood agar at 37 °C with 5 % CO_2_ for 24 h. Bacterial DNA was extracted using the ZymoBIOMICS DNA Kit (Zymo Research, Irvine, CA, USA) following the manufacturer’s instructions. Sequencing libraries were prepared using Nextera XT Library preparation kit and the XT Index kit v2 Set A (Illumina, San Diego, CA, USA). Quantity and average sizes of the libraries were measured using Qubit dsDNA assay kit (Thermo Fisher Scientific Corporation, Eugene, OR, USA) and Agilent High Sensitivity DNA kit (Agilent Technologies, Santa Clara, CA, USA), respectively. The pooled libraries were subjected to a single run of 300 bp paired-end Illumina MiSeq sequencing using the Reagent Kit v3 at Mahidol-Oxford Tropical Medicine Research Unit (MORU), Faculty of Tropical Medicine, Mahidol University. For bioinformatics analyses, default settings were used unless noted otherwise. Raw Illumina reads were filtered using fastp v0.20.0 [31] and read quality was assessed with FastQC (v0.11.8, https://github.com/s-andrews/FastQC). Filtered reads were taxonomically classified using Kraken2 (v2.0.8) and the MiniKraken v2 database to evaluate the presence of contaminant bacterial and human genomic DNA [32]. The serotype of the strains was determined using the *S. suis* serotyping pipeline fed with the filtered reads [33]. The whole-genome average nucleotide identity (ANI) of the filtered reads was assessed using FastANI v1.32 against eight complete *S. suis* genomes and one complete *Streptococcus parasuis* genome to confirm the species classification of the isolates (Table S1) [34].

To generate complete genomes, nine outbreak isolates were selected for additional long read sequencing using the Oxford Nanopore Technologies (ONT) platform. Isolates were randomly selected, stratified by gender and including the index case (STC78) for ONT long read sequencing. The rapid barcoding protocol was followed for ONT-based DNA sequencing using the SQK-RBK004 kit without selecting DNA size to preserve plasmid DNA. The libraries were sequenced using a single R9.4.1/FLO-MIN106 flow cell on a MinION Mk1B sequencer. Raw data was demultiplexed using Guppy v3.4.5 (ONT), specifying the high-accuracy model (-c dna_r9.4.1_450bps_hac.cfg). The ONT adapters were trimmed using Porechop (v0.2.4 https://github.com/rrwick/Porechop) and filtered using Filtlong (v0.20.0, https://github.com/rrwick/Filtlong) with a minimum read length of 500 bp. NanoPlot (v1.28.1 https://github.com/wdecoster/NanoPlot) was used for quality control of the ONT long reads. Draft genomes were assembled from Illumina sequencing data using SPADes v3.14.1 [35] with Shovill (v1.0.9 https://github.com/tseemann/shovill). Hybrid assemblies of ONT and Illumina data were generated using Unicycler v0.4.8 [36], and the quality of the complete genome sequences was checked using QUAST v5.0.2 [37]. The assembly graphs the bacterial chromosome and plasmids generated with Unicycler were visualized with Bandage v0.8.1 [38]. Draft and complete genomes were annotated using Prokka v1.14.6 [39].

### Phylogenetic analysis

A genome-wide single nucleotide polymorphism (SNP) based phylogeny was reconstructed to describe the evolutionary relationships of the outbreak and post-outbreak isolates using Snippy (v4.6.0, https://github.com/tseemann/snippy). In brief, filtered Illumina MiSeq reads were mapped to the complete genome of the outbreak’s index case isolate STC78. Mapping coverage was evaluated with minimap2 v2.17 [40] and samtools v1.13 [41] and three isolates (STC127, STC129 and STC154) with <90 % coverage were excluded from the phylogeny to obtain a higher resolution tree. A maximum likelihood (ML) phylogeny was inferred from 15367 core SNPs using IQ-TREE v2.0.3 [42] with 1000 bootstraps. The number of constant sites in the whole genome alignment was calculated using snp-sites v2.5.1 with the ‘-C’ flag and provided to IQ-TREE using the ‘-fconst’ flag [43]. The SYM model with ascertainment bias correction (ASC) was chosen as the optimal tree model using ModelFinder [44] and the final ML phylogenetic tree was corrected to account for recombination using ClonalFrameML [45].

A dataset containing 1703 curated *S. suis* genomes [46] was used in combination with genomes generated in this study to reconstruct the phylogeny of the global *S. suis* population (Table S1). The core genome and pangenome were inferred by clustering genes from the annotated draft assemblies into homology groups using Roary v3.13.0 [47]. The core genome alignment consisting of 469 protein-coding genes was fed into IQ-TREE v2.0.3 [42] and snp-sites v2.5.1 [43] using the GTRGAMMA model with 1000 bootstraps to generate a ML phylogenetic tree. To obtain a higher resolution tree describing the emergence of the outbreak strain, genomes from the CC104 and CC233/379 clades, including the outbreak, post-outbreak and public strains with available sequencing reads, were used to reconstruct (as previously stated) a recombination stripped genome-wide SNP phylogeny using the complete genome of the index outbreak isolate STC78 as a reference (Table S1). All trees were visualized using the interactive tree of life (iTOL) v6.5 [48].

### Acquisition of mobile genetic elements and antimicrobial resistance (AMR) genes

The presence of AMR genes was scanned in the genomic assemblies of the outbreak strains clade using AMRFinderPlus v3.10.16 [49]. To assess penicillin susceptibility, the protein sequences of the penicillin binding proteins (PBPs) were extracted and screened for amino acid substitutions known to correlate with decreased penicillin susceptibility in streptococci [50]. In brief, the sequence of each PBP was aligned using muscle v3.8.1551 [51] and visualized in SEAVIEW v5.0.5 [52]. The prokka annotations of the Thai zoonotic clade were queried to identify acquired resistance genes in the outbreak strain using Panaroov1.2.9 [53]. In addition, the STC78 complete genome was scanned for integrative and conjugative elements (ICEs) and prophages using ICEFinder [54] and PHASTER [55], respectively. Acquired AMR genes and their genomic context were manually inspected using Artemis v18.1.0 [56]. PubMed was searched for primary research articles describing mobile genetic elements (MGEs) carrying the same AMR genes to identify putative homologous MGEs. The annotated MGEs were aligned using clinker and clustermap.js v.0.021 [57]. The plasmid acquired by the outbreak strain was visualized using ApE v3.0.8 [58] and a blastn [59] search was performed against bacterial reference genomes. To assess the presence of potential genes of interest, ABRicate (https://github.com/tseemann/abricate) was used with a custom database containing the sequences of 52 genes previously found to be putatively associated with zoonotic potential of *S. suis* strains [46].

### Antimicrobial susceptibility testing

The minimum inhibitory concentrations (MICs) of penicillin, ceftriaxone, levofloxacin, linezolid, chloramphenicol, clindamycin, erythromycin and tetracycline were determined using E-Tests (Liofilchem, Abruzzi, Italy) following the supplier’s instructions and using the standards defined in the Clinical and Laboratory Standard Institute (CLSI) guidelines 2021 (M100-31^st^ edn) to interpret the results. Since there are currently no breakpoints recommended for *S. suis *, breakpoints for viridans group streptococci were used. *Streptococcus pneumoniae* strain ATCC 49619 was used for quality control purposes.

## Results

### Epidemiological investigation of the outbreak

**Fig. 1.**
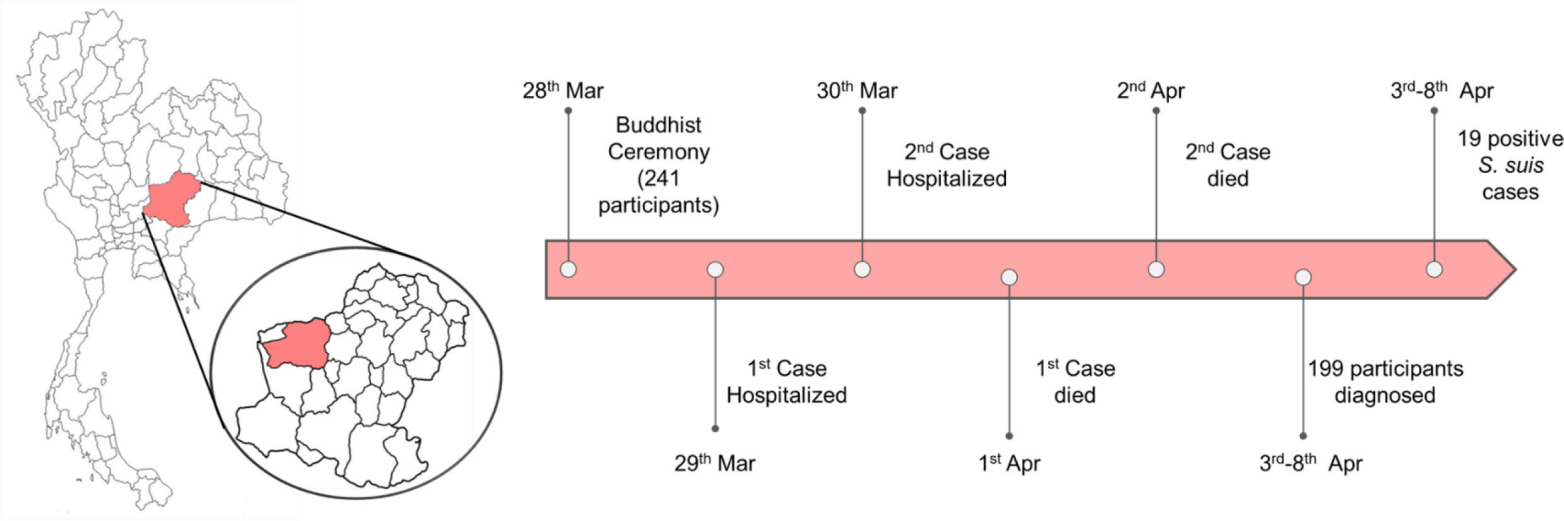
Overview of the *S. suis* Nakhon Ratchasima outbreak. The Nakhon Ratchasima province and the Dan Khun Thod district where the outbreak took place are highlighted in red. In total, 199 participants of the Buddhist ordination ceremony were examined at community hospital following local news of the two deaths caused by *S. suis *. Patient information was assessed by a team of epidemiologists from the Ministry of Public Health. The patients were also interviewed and questionnaires were conducted by the epidemiologists. From this, 88 participants were suspected cases of the outbreak and hemocultures were taken from them. Lumbar punctures were performed on suspected cases showing symptoms of meningitis (*n*=3) to collect cerebrospinal fluid (CSF). Nineteen cases were confirmed with positive cultures (hemocultures and CSF). Presumptive taxonomical identification of *S. suis* was carried out using Vitek-2 in the Maharaj Nakhon Ratchasima hospital as well as via a previously described PCR typing method [27, 28].

The outbreak occurred following a Buddhist ordination ceremony on 28 March 2021, attended by 241 participants from three villages in the Dan Khun Thod district, Nakhon Ratchasima (Fig. 1). Rice noodles with fish curry sauce, winter melon soup with pork, eggplant curry with pork, sour and sweet stir-fry with pork, and spicy minced raw pork were served during the ceremony to all guests.

On March 29^th^, the first case, a 67-year-old male who had joined the ceremony, was admitted to the community hospital after presenting with high fever, chills, vomiting, and diarrhoea symptoms. On March 30th, he was referred to the Maharaj Nakhon Ratchasima hospital, where his condition deteriorated. On March 31^st^, he manifested purpura throughout his body, septic shock, loss of consciousness, and acute renal failure. This first case passed away on April 1^st^. The second case was a 73-year-old male; this case did not join the ceremony but ate spicy minced raw pork which his wife had brought home from the ceremony on March 28^th^. He was admitted to the community hospital on March 30^th^ with high fever, chills, vomiting, and dyspnea and was referred to the provincial hospital on the same day. On April 2^nd^; he passed away from septic shock. The deaths of these two cases prompted participants of the ceremony to visit the community hospital; and between April 3^rd^ and April 8^th^, 199 people were examined. Among these, 88 were suspected cases and 19 were confirmed by positive hemoculture.

Epidemiologists from the Nakhon Ratchasima Public Health Office found the spicy minced raw pork dish to be the most probable cause of infection in this outbreak, and the source of the pork was investigated [26]. The pork originated from a medium size contract farm. Contract farms raise pigs for larger companies and generally follow strict guidelines on antibiotic usage. The pork was slaughtered and stored without refrigeration in a local slaughterhouse and was bought for the ceremony from a shop in a wet market in the district. No pork samples from the ceremony could be recovered during the investigation.

### Clinical manifestations

The majority of patients were male (57.9 %; 11/19) and the median age was 67 years (range 33–89 years). All cases had a history of spicy minced raw pork consumption at the ordination ceremony. Clinical symptoms ranged from mild to severe including fever, headache, stiff neck, diarrhoea, dyspnoea, joint pain, back pain, and purpura. Sepsis was the predominant manifestation in this outbreak occurring in 84.2 % (16/19) of the cases, whereas three cases presented with meningitis, one of which with septic arthritis as well. Disseminated intravascular coagulation (DIC) was found in the index case, STC78 (Table 1). Two patients died from septic shock. Hearing loss was not reported. Patients were treated with ceftriaxone for 14 days.

**Table 1.** Clinical manifestations of 19 outbreak cases and seven post-outbreak cases. M stands for male and F stands for female. CKD stands for chronic kidney disease. na stands for not available

Case information								Clinical information					
Case ID	Note	Sex	Age (years)	Admitted	Discharged	Location (District)	History of raw pork consumption	Clinical symptom	Diagnosis	Complications	Comorbidity	Treatment	Outcome
STC78	Outbreak	M	67	Mar 29^th^	Apr 1^st^	Dan KhunThod	Yes	Fever, dyspnoea, diarrhoea, purpura	Sepsis	Disseminated intravascular coagulation	None	Ceftriaxone and meropenem	Died (Septic shock)
STC79	Outbreak	M	73	Mar 30^th^	Apr 2^nd^	Dan KhunThod	Yes	Fever, dyspnoea, diarrhoea	Sepsis	None	Alcohol abuse	Ceftriaxone and meropenem	Died (Septic shock)
STC80	Outbreak	F	72	Mar 30^th^	Apr 1^st^	Dan KhunThod	Yes	Fever, headache	Sepsis	None	None	Ceftriaxone	Alive
STC81	Outbreak	M	68	Mar 30^th^	Apr 12^th^	Dan KhunThod	Yes	Fever	Sepsis	None	Alcohol abuse and diabetes mellitus	Ceftriaxone	Alive
STC82	Outbreak	M	79	Mar 31^st^	Apr 17^th^	Dan KhunThod	Yes	Fever, headache	Sepsis	None	None	Ceftriaxone	Alive
STC83	Outbreak	M	75	Apr 3^rd^	Apr 16^th^	Dan KhunThod	Yes	Fever	Sepsis	None	Hypertension	Ceftriaxone	Alive
STC84	Outbreak	F	62	Apr 1^st^	Apr 6^th^	Dan KhunThod	Yes	Fever	Sepsis	None	Hypertension	Ceftriaxone	Alive
STC85	Outbreak	F	50	Apr 4^th^	Apr 7^th^	Dan KhunThod	Yes	Fever, headache, backpain	Sepsis	None	Hypertension and diabetes mellitus	Ceftriaxone	Alive
STC86	Outbreak	M	64	Apr 2^nd^	Apr 3^rd^	Dan KhunThod	Yes	Fever, diarrhoea	Sepsis	None	Alcohol abuse	Ceftriaxone	Alive
STC87	Outbreak	M	33	Apr 3^rd^	Apr 6^th^	Dan KhunThod	Yes	Fever, diarrhoea	Sepsis	None	Alcohol abuse	Ceftriaxone	Alive
STC88	Outbreak	M	67	Apr 4^th^	Apr 6^th^	Dan KhunThod	Yes	Fever, joint pain	Sepsis	None	Alcohol abuse	Ceftriaxone	Alive
STC89	Outbreak	M	73	Apr 4^th^	Apr 5^th^	Dan KhunThod	Yes	Fever, headache, joint pain	Meningitis	None	Alcohol abuse and hypertension	Ceftriaxone	Alive
STC90	Outbreak	F	66	Apr 3^rd^	Apr 17^th^	Dan KhunThod	Yes	Fever, headache	Sepsis	None	None	Ceftriaxone	Alive
STC91	Outbreak	F	66	Apr 3^rd^	Apr 17^th^	Dan KhunThod	Yes	Fever, headache	Sepsis	None	Hypertension	Ceftriaxone	Alive
STC92	Outbreak	F	72	Apr 3^rd^	Apr 17^th^	Dan KhunThod	Yes	Fever	Sepsis	None	None	Ceftriaxone	Alive
STC93	Outbreak	M	89	Apr 3^rd^	Apr 17^th^	Dan KhunThod	Yes	Fever, stiff neck	Meningitis	None	Alcohol abuse, bronchiectasis	Ceftriaxone, ampicillin	Alive
STC104	Outbreak	F	67	Apr 5^th^	Apr 18^th^	Dan KhunThod	Yes	Fever, stiff neck, back pain, joint pain	Meningitis Septic arthritis	None	Hypertension, CKD	Ceftriaxone, cefixime	Alive
STC105	Outbreak	M	64	Apr 6^th^	Apr 7^th^	Dan KhunThod	Yes	Fever, headache	Sepsis	None	None	Ceftriaxone	Alive
STC106	Outbreak	M	64	Apr 5^th^	na	Dan KhunThod	Yes	Fever, headache	Sepsis	None	Hypertension	Ceftriaxone	Alive
STC127	Post-outbreak	M	67	May 21^st^	na	Dan KhunThod	Yes	na	Sepsis, spondylodiscitis	None	None	Ceftriaxone	Alive
STC129	Post-outbreak	M	80	May 24^th^	na	Dan KhunThod	Yes	na	Sepsis, meningitis	None	None	Ceftriaxone	Alive
STC130	Post-outbreak	M	48	May 25^th^	na	Khong	Yes	na	Sepsis	None	Hypertension, diabetes mellitus	Ceftriaxone	Alive
STC131	Post-outbreak	M	46	May 26^th^	na	Dan KhunThod	Yes	na	Sepsis, endocarditis	None	None	Ceftriaxone	Alive
STC152	Post-outbreak	M	67	May 23^rd^	na	Non Sung	Yes	na	Meningitis	None	Hypertension	Ceftriaxone	Alive
STC153	Post-outbreak	na	na	na	na	na	na	na	na	None	na	na	na
STC154	Post-outbreak	M	55	May 30^th^	na	Sung Noen	Yes	na	Spondylodiscitis	None	Cirrhosis	Penicillin G	Alive

Seven post-outbreak cases of *S. suis* infection were identified in a follow-up investigation in Nakhon Ratchasima during May, 2021. Sepsis was the most common manifestation (66 %; 4/6, clinical data missing for one post-outbreak case). Additionally, meningitis and spondylodiscitis were observed in two patients and endocarditis in one patient (Table 1).

### The outbreak was caused by a single clone belonging to a zoonotic *S. suis* lineage emerging in Thailand

The initial *S. suis* taxonomic identification was confirmed through average nucleotide identity (ANI) analysis of the 22 sequenced isolates, showing >96 % ANI to eight complete *S. suis* genomes (Table S2). All outbreak and post-outbreak isolates were identified as serotype 2 using the filtered reads and the *S. suis* serotyping pipeline [33]. Multi-locus sequence typing (MLST) showed that the outbreak isolates were of a novel sequence type (ST) ST1656, belonging to the zoonotic clade CC233/379 which emerged in Thailand in the last two decades [22]. Post-outbreak isolates were identified as ST104 (*n*=1), ST233 (*n*=2), ST379 (*n*=1) and novel ST1688 (*n*=3), all belonging to the Thai zoonotic lineages CC104 and CC233/379, with the exception of ST1688 isolates which did not form part of any previously reported CCs [22] (Fig. S1).

**Fig. 2.**
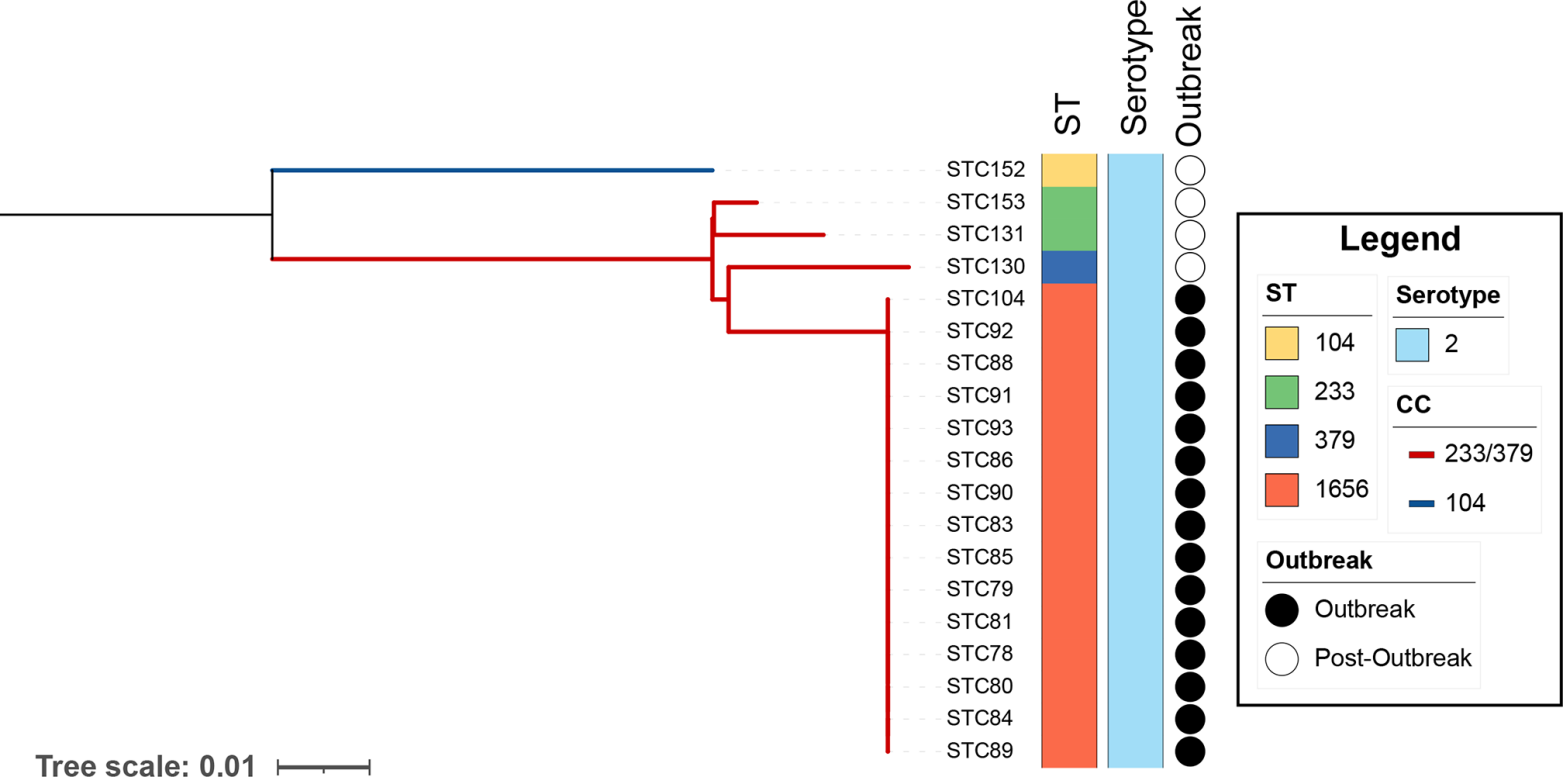
SNP reference mapped phylogeny of outbreak and post-outbreak *S. suis* isolates. The maximum-likelihood phylogeny was reconstructed using Snippy and IQ-TREE by mapping the filtered Illumina reads of each isolate to the complete genome of the index case (STC78). Branch lengths were corrected for recombination using ClonalFrameML. Outbreak isolates are marked with a black circle. Information regarding the ST and serotype of each isolate is included in the columns adjacent to the phylogenetic tree. The two lineages CC104 and CC233/379 are coloured blue and red respectively in the tree.

The whole-genome single nucleotide polymorphism (SNP) based phylogenetic tree showed that all 15 outbreak isolates clustered together and had no SNPs compared to the reference (index case STC78), confirming that the outbreak was caused by a single *S. suis* clone. The outbreak clone diverged from other CC233/379 isolates, differing by 3398 and 2615 SNPs from the ST233 strains STC131 and STC153 respectively, and by 4030 SNPs from the ST379 strain STC130 (Fig. 2). The outbreak clone contained 29 out of 52 zoonosis associated genes (Fig. S2), including suilysin (*sly*) and enolase (*eno*), both well-studied virulence associated factors [46]. However, the outbreak clone lacked both muramidase-released protein (*mrp*) and factor H binding protein (*fhb*) which have been reported to facilitate attachment and translocation across the Blood Brain Barrier (BBB) [60, 61] (Fig. S2). Furthermore, the outbreak clone contained an unclassified 4.2 kb plasmid pSTC78 containing two hypothetical proteins (Fig. S3; Table S3). The pSTC78 plasmid appears to be mostly *S. suis* specific as 95.6 % (65/68) of the nucleotide blast hits on bacterial reference genomes were *S. suis* genomes (Fig. S4).

### Thai zoonotic lineages have emerged independently from the main *S. suis* zoonotic clades

**Fig. 3.**
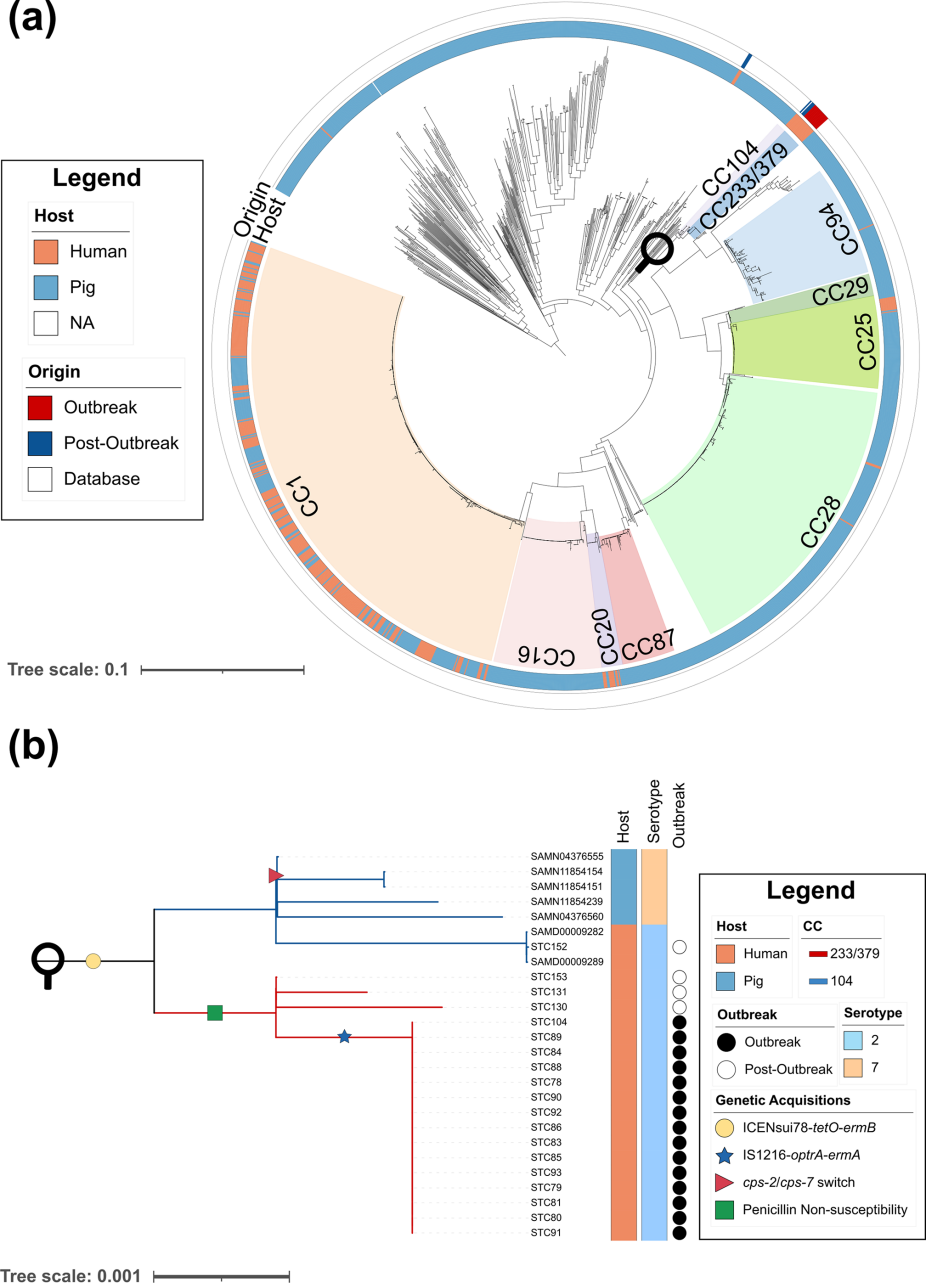
Emergence of outbreak clone within the context of the global *S. suis* population. (**a**) Core-genome maximum-likelihood phylogeny based on 1725 *S *. *suis* genomes reconstructed using Roary and IQ-TREE. Inner ring represents the host from which strains were isolated. The outer ring indicates whether strains were collected during this study or were part of a previously curated *S. suis* dataset [46]. The main *S. suis* CCs are annotated with various colours. The magnifying lens indicates the clade to which the outbreak strain belongs. (**b**) Whole genome SNP reference mapping based phylogeny of the outbreak and related clades reconstructed using Snippy and IQ-TREE. Branch lengths were corrected for recombination using ClonalFrameML. Support values for each node of the genome-wide SNP based tree used as an input for ClonalFrameML are shown in Fig. S5. The seven additional CC104 isolates were isolated from human (*n*=2) and pig (*n*=5). Human isolates were isolated in Thailand whereas the porcine isolates were isolated in the USA (*n*=3), China (*n*=1), and Canada (*n*=1) [33, 76]. Columns adjacent to the tree contain information regarding the host, source, ST and serotype for each isolate. The two different CCs are coloured in blue (CC104) and red (CC233/379) in the tree. The yellow circle indicates the acquisition of ICENsui78te. The blue star indicates the acquisition of the IS1216-*optra-ermA*. The red triangle indicates a capsule switching event. The green square indicates progressive acquisition of mutations in the PBPs.

To understand the emergence of the outbreak clone, a core-genome SNP based phylogeny of 1703 global *S. suis* genomes and outbreak isolates was made. Both CC104 and CC233/379 isolates clustered together, forming a Thailand specific zoonotic clade. The Thai zoonotic CCs were distantly related to the other zoonotic lineages (CC1, CC20 and CC25), suggesting that adaptations which would allow them to cause symptomatic infections in humans may have occurred independently in virulent porcine lineages (Fig. 3a). To obtain a higher resolution of the Thai zoonotic clade, the available sequencing reads from the CC104 and CC233/379 isolates were used to build a second genome-wide SNP based phylogeny specific for this clade (Fig. 3b). CC104 and CC233/379 clustered into two separate clades and the topology of the tree was well supported (Fig. S5). Within CC104, the three zoonotic isolates clustered together in a sub-lineage separate from the porcine strains. Interestingly, all zoonotic strains in CC104 were serotype 2 while all porcine strains were serotype 7, indicating that capsule switching events have occurred during the evolution of the CC104.

### The outbreak clone acquired multiple drug resistance determinants

**Fig. 4.**
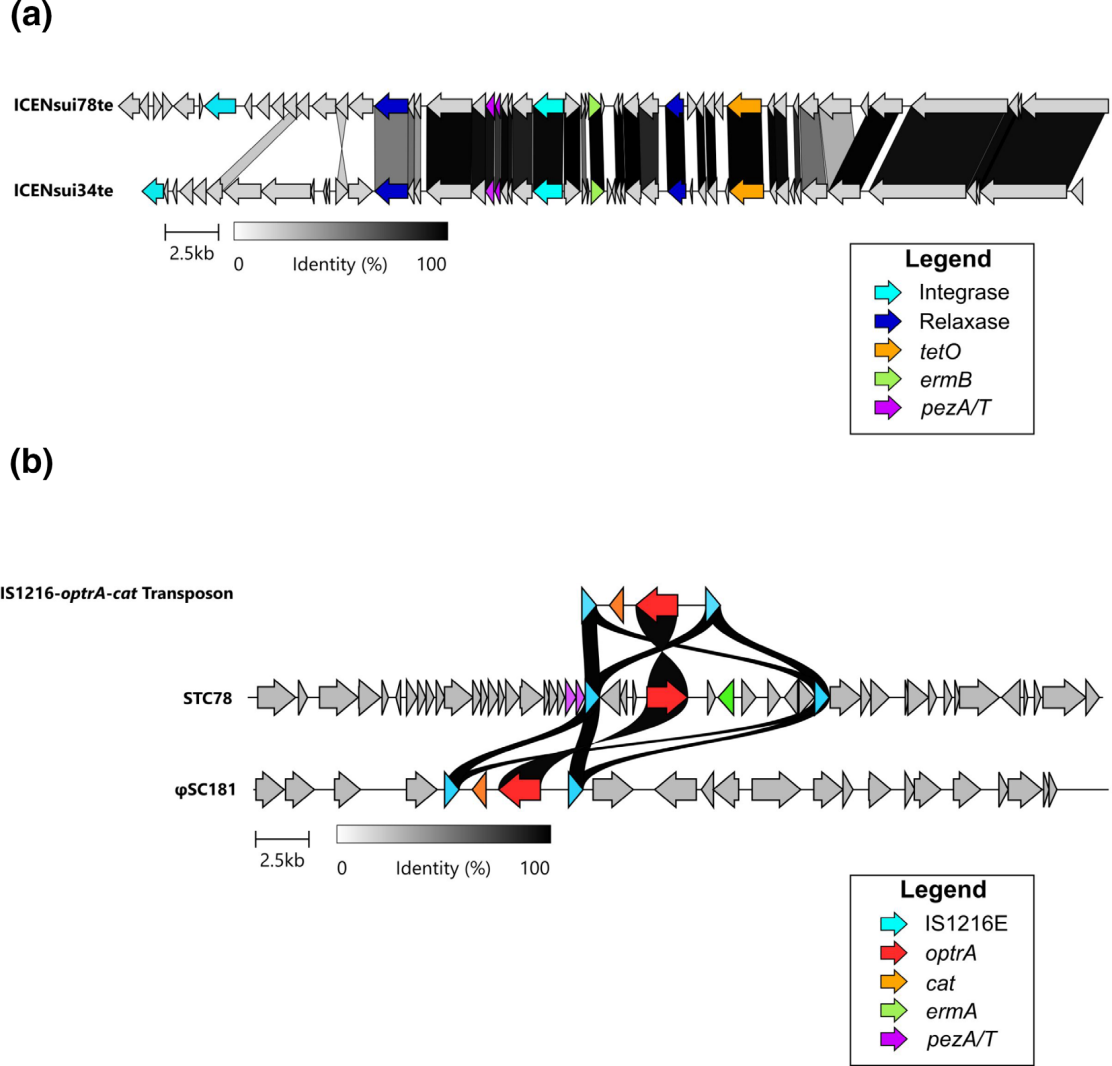
MGEs containing AMR genes acquired by the outbreak clade. (**a**) Protein level alignment of the ICENsui78te with the ICENsui34te carrying *tetO* and *ermB* previously found in strains isolated in Thailand [62]. The ICENsui34te annotated sequence was extracted from strain DP_SS29 2 (NZ_WCIZ01000002.1 [63]). (**b**) Protein level alignment of the IS1216 Transposon with the φSC81 prophage (MK359990.1 [66]) and the STC78 genomic region where it was inserted. Genes of interest are coloured and defined in the legend. Alignments were made using clinker and clustermap.js.

Antimicrobial resistance (AMR) markers were screened for in the CC104 and CC233/379 strains using AMRfinderPlus. Tetracycline resistance gene *tetO* and macrolide–lincosamide–streptogramin B (MLS_B_) resistance gene *ermB* were found in all strains from the CC104 and CC233/379 clades. Inspection of the STC78 genome revealed that *tetO* and *ermB* were localized closely together (7035 kb apart) on a putative ICE identified by ICEfinder, which we named ICENsui78te (Table S3; ON944185). Previous investigations reported acquisition of *tetO* and *ermB* in Thai *S. suis* isolates via the ICENsui34te mobile genetic element [62]. To compare ICE composition, we aligned ICENsui78te to ICENsui34te found in strain DP_SS29 2 (NZ_WCIZ01000002.1 [63]); (Fig. 4a). Large regions of both ICEs were highly similar (>95 % amino acid identity), however, ICENsui78te encoded a different putative integrase (Fig. 4a). Additionally, ICENsui78te contained the *pezAT* Toxin/Antitoxin system, which is presumed to promote integration of mobile genetic elements and is commonly found in mobile genetic elements in the *Streptococcus* genus, including *S. suis* [64].

MLS_B_ resistance gene *ermA,* and oxazolidinone and phenicol resistance gene *optrA* were found exclusively in the outbreak clone (Fig. 3b). Both genes colocalized in the STC78 genome in a region identified as a putative mobile genetic element by ICEfinder and PHASTER. IS1216 insertion sequences flanked both genes. IS1216 transposons have been associated with *optrA* mobility across *S. suis* in the past. Previous studies observed that multiple *S. suis* strains had acquired *optrA* through prophages and ICEs carrying a IS1216 transposon [65, 66]. To examine the homology between the IS1216-*optrA-ermA* transposons, we aligned the STC78 genomic region containing the putative IS1216 against the previous reported *optrA* harbouring mobile genetic elements. Similarity was only found between the IS1216 transposon carrying *optrA* (Fig. 4b). Additionally, no putative integrases, relaxases or phage genes were identified near the IS1216-*optrA-ermA* transposon in the STC78 genome. Instead, a second copy of the *pezAT* Toxin/Antitoxin system was found directly upstream of the IS1216-*optrA-ermA* (Table S3; ON944186).

The penicillin binding protein (PBP) sequences from the Thai zoonotic clades were aligned and critical residues previously found to reduce susceptibility to penicillin were inspected [50]. The CC104 isolates harboured the susceptible PBPs alleles whereas CC233/379 had progressively accumulated key mutations in Pbp2X, Pbp2B and MraY, known to reduce susceptibility to penicillin (Table S4). ST379 and ST1656 isolates had two additional mutations compared to ST233 isolates, as only ST379 and STC1656 isolates had acquired mutations in MraY.

The susceptibility profile for eight antibiotics (penicillin, ceftriaxone, levofloxacin, linezolid, chloramphenicol, clindamycin, erythromycin and tetracycline) was determined for all 22 isolates collected in this study (Table 2). All strains were resistant to erythromycin and clindamycin, while six of the strains were resistant to tetracycline; the two susceptible isolates belonged to the novel ST1688. No strains were susceptible to chloramphenicol, the outbreak clone being resistant while post-outbreak strains had intermediate resistance. The CC233/379 strains were non-susceptible to penicillin while the ST1688 isolates were resistant to penicillin. Only the outbreak clone was resistant to linezolid. Overall, the aforementioned acquisition events of MGEs conferred reduced susceptibility against six antibiotics from different antibiotic classes to the outbreak clone, only remaining susceptible to ceftriaxone and levofloxacin.

**Table 2.** Minimum inhibitory concentration of eight antibiotics against *S. suis *. Antimicrobial susceptibility was tested through using E-tests. Breakpoints from viridans group *Streptococci* described in the CLSI 2021 guidelines were used and are indicated below each antibiotic. (S)=Susceptible, (I)=Intermediate and (R)=Resistant. The range of the MIC values for the 15 outbreak isolates are shown under the STC78 index case

			MICs							
Strain	Note	ST	Penicillin (μg ml^−1^) S=≤0.12 I=0.2–0.25 R=≥4	Ceftriaxone (μg ml^−1^) S=≤1 I=2 R=≥4	Levofloxacin (μg ml^−1^) S=≤2 I=4 R=≥8	Linezolid (μg ml^−1^) S=≤2	Chloramphenicol (μg ml^−1^) S=≤4 I=8 R=≥16	Clindamycin (μg ml^−1^) S=≤0.25 I=0.5 R=≥1	Erythromycin (μg ml^−1^) S=≤0.25 I=0.5 R=≥1	Tetracycline (μg ml^−1^) S=≤2 I=4 R=≥8
STC78	Outbreak	1656	0.5–0.75	0.75–1	0.125–0.19	3–8	12–24	>256	>256	16–32
STC127	Post-outbreak	1688	4	0.25	0.19	1	6	>256	>256	16
STC129	Post-outbreak	1688	4	0.75	0.5	1	8	>256	>256	0.125
STC130	Post-outbreak	379	0.5	1	0.19	0.75	6	>256	>256	24
STC131	Post-outbreak	233	0.5	1	0.19	0.75	6	>256	>256	32
STC152	Post-outbreak	104	0.094	0.05	0.19	1	6	>256	>256	12
STC153	Post-outbreak	233	0.5	0.75	0.19	1	8	>256	>256	16
STC154	Post-outbreak	1688	4	1	0.75	1	6	>256	4	0.125

## Discussion

Since 2001, four outbreaks of *S. suis* infections in humans have been recorded in northern Thailand [19]. The first outbreak occurred in Phayao in May 2007 and resulted in 29 confirmed cases, three of which were fatal [67]. A second outbreak was reported in Chiang Mai and Lamphun in June–July 2008, including 44 confirmed cases, and three fatal cases from septic shock [19]. These two outbreaks were both caused by serotype 2 ST1 *S. suis* strains (unpublished data). The third outbreak occurred in Phetchabun in April 2010 with 19 confirmed cases and five deaths, and was caused by strains of both serotype 14 ST105 (CC1) and serotype 2 ST104 (CC104) [19]. The fourth outbreak was reported in Uttaradit province in May 2019 with 14 cases with infection by *S. suis* of unknown serotype and ST [19]. For the outbreaks in 2007 and 2010 a case control study found the consumption of raw pork products as the most probable cause of the outbreaks [19, 67]. However, outbreaks remained uncharacterized at a genomic level. In this study, we described a fifth *S. suis* outbreak in Thailand by combining clinical, epidemiological and genomic data.

The 2021 Nakhon Ratchasima outbreak is the first reported outbreak caused by *S. suis* in Northeast Thailand as well as the first reported outbreak caused by the Thai clade CC233/379 (Fig. 2). Although CC1 is distributed worldwide and accounts for most human infections, no CC1 isolates were recovered in this study. Instead, all outbreak and post-outbreak isolates belonged to zoonotic clades that originated in Thailand (CC104, CC233/379 and ST1688), suggesting that at the time of the outbreak, zoonotic strains from Thai lineages were the most common strains circulating in pigs in Dan Khun Thod district. Isolates from the Thai zoonotic lineages CC104 and CC233/379 accounted for 37.1 % (248/668) of previously reported human infections in Thailand, however, there have been no genomic studies of the Thai *S. suis* population investigating how these clades acquired zoonotic potential [22, 23].

Acquisition of a serotype 2 capsule may be one of the critical determinants contributing to increased survival of *S. suis* during human infections. Within the global *S. suis* phylogeny, we observed evidence of capsule switching between serotype 2 and 7 across human and porcine isolates of CC104. Similarly, the emergence of the CC20 zoonotic clade in the Netherlands was pre-dated by capsule switching events between *S. suis* strains of serotypes 9, 4 and 2 [21]. While the mechanistic advantage of the serotype 2 capsule over other serotypes for human infection remains unclear, there is a clear association between human infections and serotype 2, amounting up to 97.0 % (1227/1265) of the human infections with reported serotypes as opposed to 27.1 % (1331/4711) in pig infections [20].

The main clinical manifestation of the outbreak cases was sepsis (84.2 %; 16/19) followed by bacterial meningitis (15.8 %; 3/19) (Table 1), which is in contrast with previous systematic studies of endemic infections where meningitis was reported as the primary clinical manifestation during *S. suis* infections with a pooled rate of 68 % [4]. However, clinical manifestations may be influenced by the characteristics of strains circulating in certain geographic locations. For instance, in a study including 668 Thai human *S. suis* infections collected between 2009 and 2012 across 39 provinces, sepsis was observed in 75 % (501/668) of patients whereas meningitis only accounted for 21 % of clinical manifestations (141/668) [22]. Furthermore, in this study an association between the clinical manifestations and CC was reported; most of the meningitis cases were caused by CC1 strains (83.7 %; 118/141) whereas meningitis was mostly absent from the cases infected with *S. suis* belonging to the Thai emergent zoonotic clades CC104 (9.9 %; 21/212) and CC233/379 (5.5 %; 2/36) [22].

Antimicrobial susceptibility testing revealed that the outbreak clone is multi-drug resistant, with acquired resistance against tetracycline, erythromycin, clindamycin and chloramphenicol as well as non-susceptibility against penicillin and linezolid, whilst only being susceptible to levofloxacin and ceftriaxone (Table 2). A previous study showed that resistance to tetracyclines and MLS_B_ is widespread amongst zoonotic *S. suis* isolated in Thailand, whereas levofloxacin and ceftriaxone remain highly effective against *S. suis* [25]. In contrast, in this study, resistance against chloramphenicol and penicillin was rare with 72.9 % (327/448) of the study’s isolates being susceptible to chloramphenicol and 91.8 % (411/448) to penicillin [25]. Furthermore, a large-scale analysis of AMR determinants in global *S. suis* solates found that only 28.0 % (190/678) and 0.4 % (3/678) of isolates had genetic determinants encoding for β-lactams (penicillin) and phenicols (florfenicol) resistance, respectively [50].

The intensification of animal farming and the increased and extensive use of antimicrobials in the last decades has led to a rise in the prevalence of AMR [68]. Penicillin is extensively used in Thai pig farms [69]. Furthermore, a study in Khon Kaen Province, Thailand reported that penicillin was administered in combination with streptomycin in all medium scale farms included in the investigation [68]. Extensive use of penicillin may have exerted favourable evolutionary pressure on PBP amino acid substitutions conferring resistance in the *S. suis* population (Table S4). Often, *S. suis* acquires AMR determinants via mobile genetic elements such as ICEs, prophages and transposons [70]. Here, we identified an ICE containing *tetO* and *ermB* in all CC233/CC104 *S. suis* isolates, named ICENsui78te (Fig. 2). ICENsui78te is highly similar to other ICE carrying *ermB* and *tetO* identified in *S. suis* ST25 strains and this family of ICE seems to have mediated the widespread acquisition of resistance against tetracyclines and MLS_B_ in Thailand (Fig. 4) [62]. Furthermore, outbreak isolates had integrated an IS1216 transposon carrying *optrA* and *ermA* conferring resistance to MLS_B_, oxazolidinone and phenicols. Presumably, this transposon was obtained via other mobile genetic elements, as similar IS1216-*optrA* transposons with the ability to form circular intermediates have been identified in multiple *S. suis* ICEs and prophages [65, 66, 71]. IS1216 transposons also play a role in disseminating *optrA* via horizontal gene transfer in other Gram-positive bacteria and multiple plasmids carrying IS1216-*optrA-*IS1216 structures have been previously described in *Enterococcus faecalis* [72, 73]. Finally, while it would have been valuable to set the outbreak clone’s acquisition events in its temporal context through a dated phylogeny, this was not possible due to the low sample size and lack of dating metadata.

In conclusion, we have characterized the Nakhon Ratchasima 2021 *S. suis* outbreak caused by a novel ST1656 strain which had acquired multiple drug resistance determinants. The emergence of such multi-drug resistant strains is alarming, especially the loss of susceptibility against penicillin as β-lactams are the preferred antibiotics to treat zoonotic *S. suis* infections [50]. Even though stricter regulations regarding antibiotic usage in farming were recently implemented in Thailand, our findings indicate that the necessary resources to enforce such regulations need to be allocated [69]. Consumption of raw pork products continue to be the predominant risk factor for *S. suis* infections in Thailand and Vietnam [1, 74, 75]. In this regard, food safety campaigns educating on the risks of consumption of *S. suis* infected pork products were shown to be effective tools to significantly reduce the incidence of *S. suis* in Thailand and should be applied periodically to prevent future outbreaks [2, 75]. Finally, active surveillance of circulating *S. suis* strains in swine and in humans, in combination with the development and use of vaccines against pathogenic *S. suis* in pigs can contribute to reducing the incidence of both porcine zoonotic infections.

## Supplementary Data

Supplementary material 1

Supplementary material 2
