# Supplementary material for: Streptococcus suis outbreak caused by an emerging zoonotic strain with acquired multi-drug resistance in Thailand
Source: Microb Genom. 2023 Feb 15;9(2):mgen000952. doi: 10.1099/mgen.0.000952 (PMC9997742; doi:10.1099/mgen.0.000952)
Supplement: Supplementary material 2 [file mgen-9-952-s001.pdf]

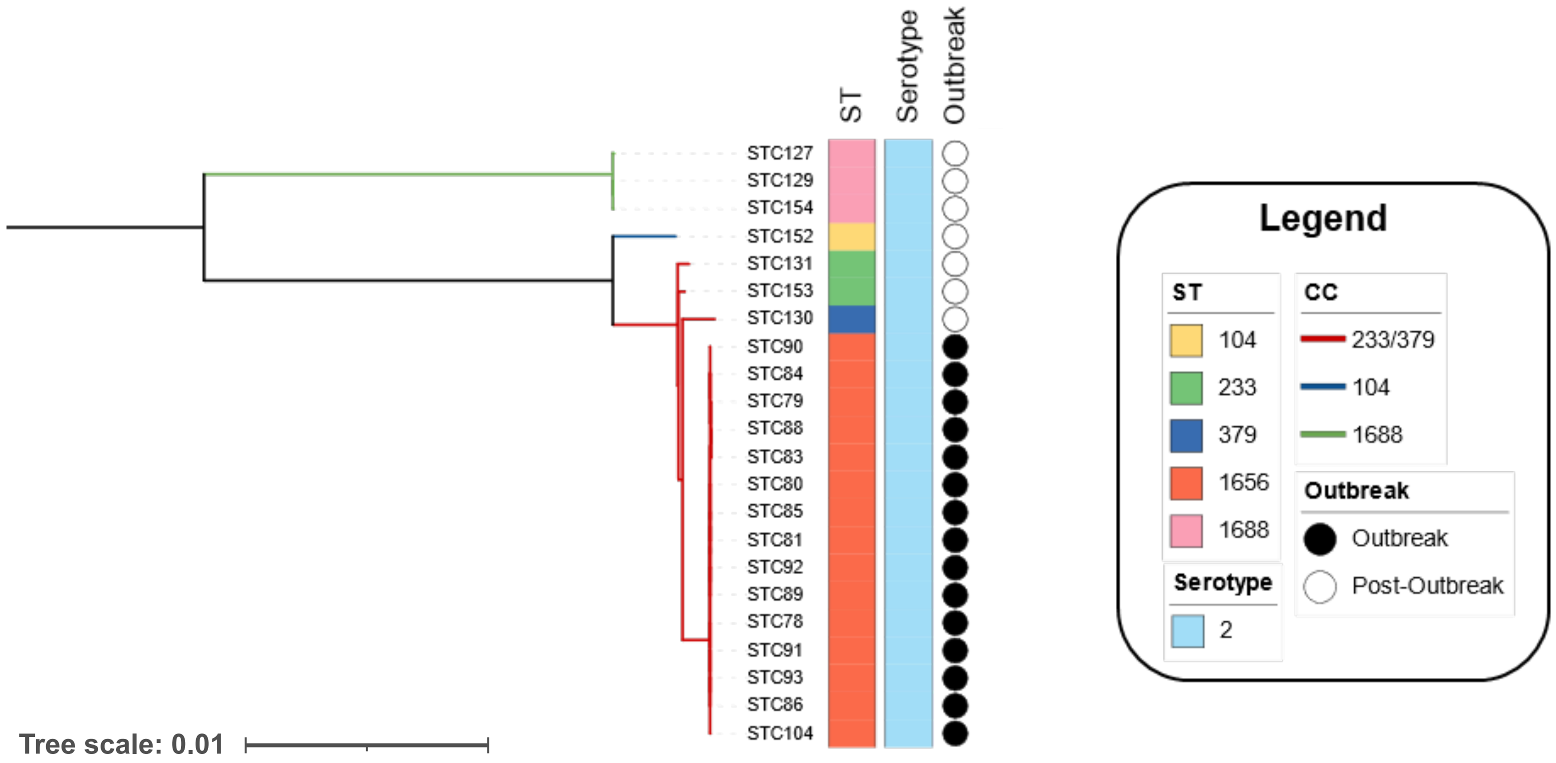


**Supplementary Figure 1.** Core-genome maximum-likelihood phylogeny (1000 bootstraps) of all isolates sequenced during this study reconstructed using Roary and iq-tree. Outbreak isolates are marked with a black circle. Information regarding the ST and Serotype of each isolate is included in the columns adjacent to the phylogenetic tree. The three different CCs are marked in green (CC1688), blue (CC104) and red (CC233/379).

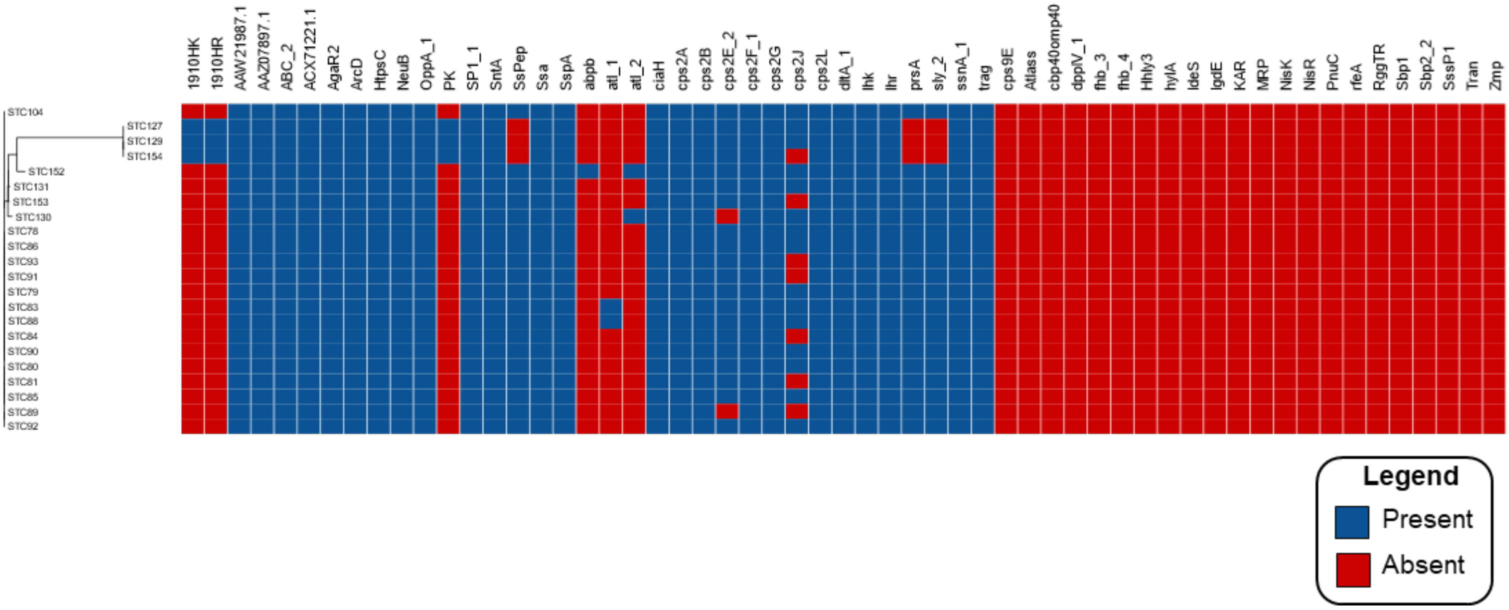


**Supplementary Figure 2.** Presence/Absence matrix of 52 potential virulence genes. The core-genome phylogeny from Supplementary Figure 1 was used. Blue squares indicate presence while red squares indicate absence of the gene.

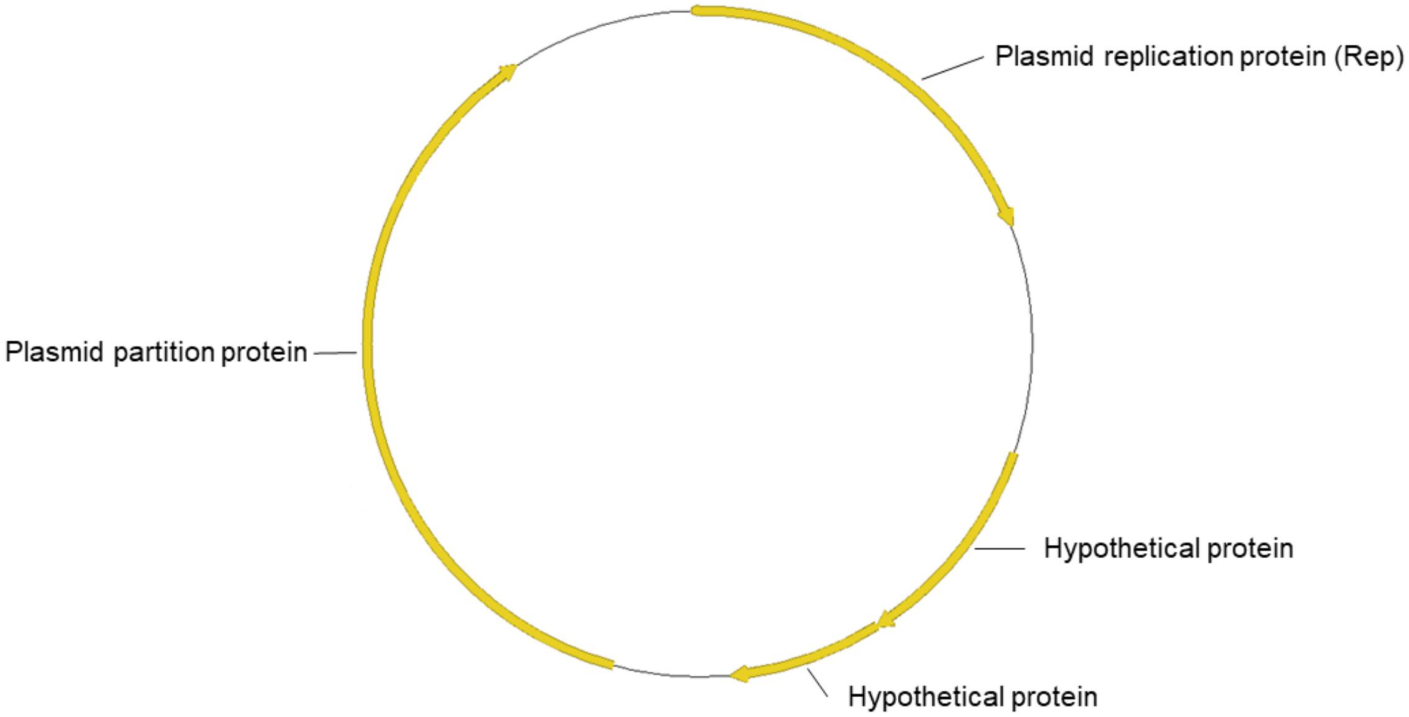


**Supplementary Figure 3. The pSTC78 plasmid found in the outbreak strains.** The sequence was extracted from the STC78 complete genome and visualized using ApE. Accession number: ON944184

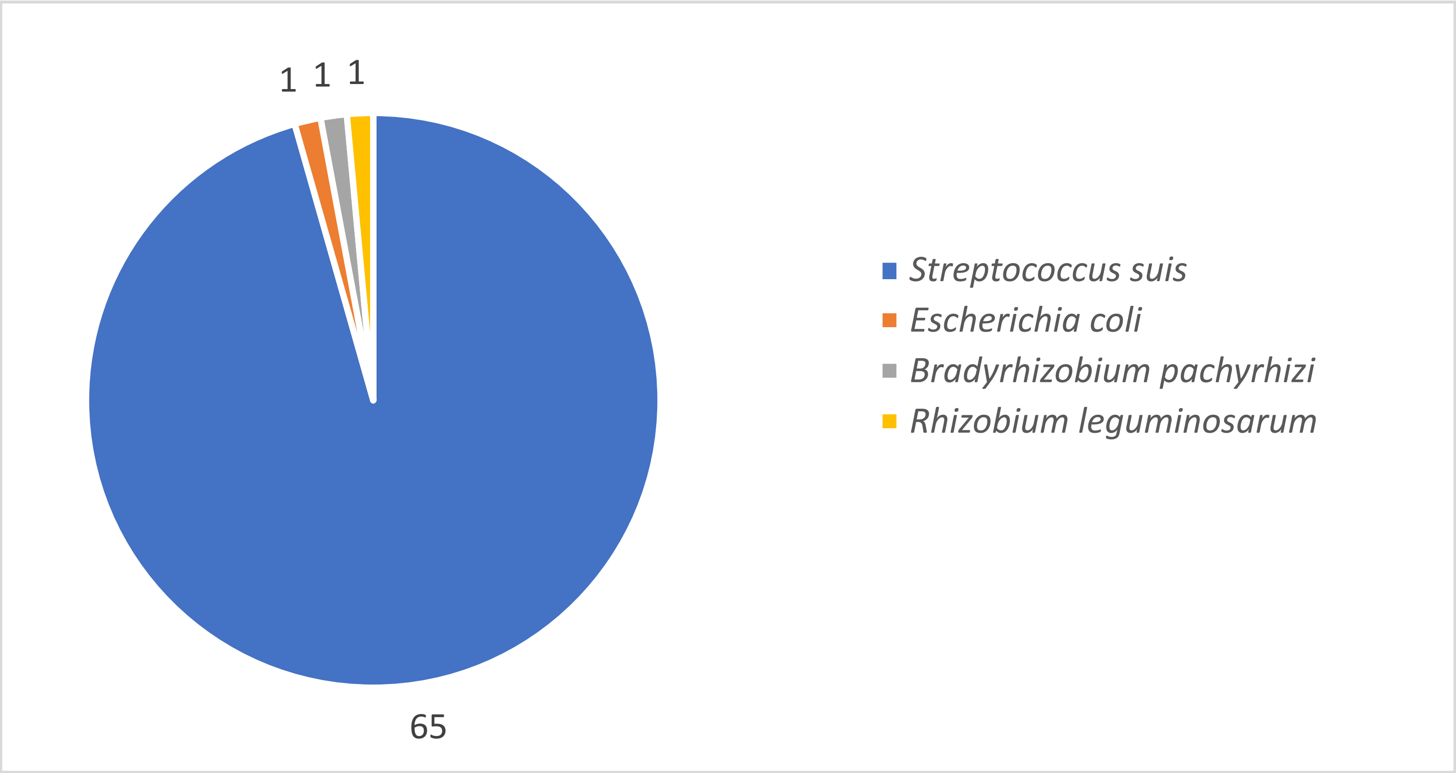


**Supplementary Figure 4. Distribution of bacterial reference genomes by species which carry the pSTC78 plasmid.** Only blast hits with >95% nucleotide identity were included.

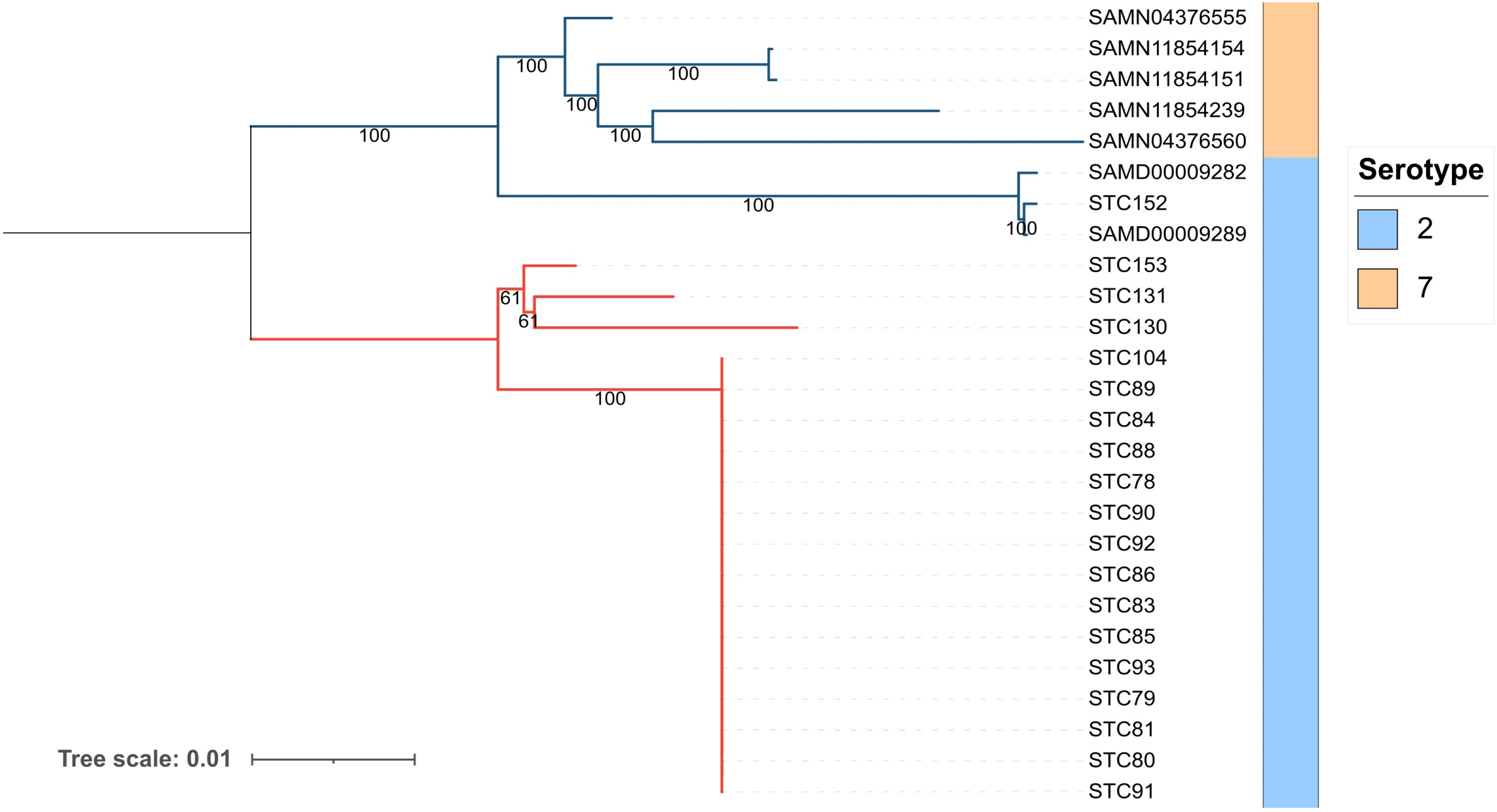


**Supplementary Figure 5.** Genome-wide SNP based phylogeny of the outbreak and related strains prior to recombination stripping. This phylogeny was used as input to generate the final recombination stripped phylogeny shown in Figure 3B. The two different CCs are coloured in blue (CC104) and red (CC233/379) in the tree. The coloured vertical column indicates Serotype. Support values are indicated for each node.
